# Preschool Children’s and Adults’ Expectations About Interpersonal Space

**DOI:** 10.3389/fpsyg.2018.02479

**Published:** 2018-12-11

**Authors:** Markus Paulus

**Affiliations:** Department of Psychology, Ludwig-Maximilians-Universität München, Munich, Germany

**Keywords:** social space, reasoning, preschool children, social distance, social cognition, action prediction

## Abstract

The regulation of interpersonal distance and social space plays a central role in social behavior, and intrusions into personal space often lead to irritations in social interactions. Although there is plenty of research on people’s actual proxemics in social interactions, less is known about how individuals represent and reason about social space, and whether there are age-related differences. The current study examined preschool children’s and adults’ predictions about others’ interpersonal distances in two experiments. The findings show that preschool children have systematic expectations about others’ proxemics. In addition, we found age-related differences as adults assumed people to keep greater interpersonal distance than preschool children.

## Introduction

In the Newtonian sense, space is a neutral parameter that is independent from the observer. It is mathematically described by three-dimensional vectors that abstract from concrete situations and contexts. Yet, for humans space and spatial position convey meaning (Low, 2003). Most notably, social interactions involve subtle, but important notions of how to position oneself with respect to another person, that is, how to regulate interpersonal distance (Hall, 1966). Interpersonal distance is regulated by a variety of factors, for example, the emotional state of the other person (Ruggiero et al., 2017; Cartaud et al., 2018), the other’s gender and age (Iachini et al., 2016), and the action abilities of the protagonist (Quesque et al., 2017). Violations of these regularities and intrusions into personal space usually lead to irritations in social interactions (Burgoon et al., 2007; Kennedy et al., 2009; Knapp et al., 2013) and aversive reactions (Evans and Wener, 2007). Notwithstanding the wide interest in how humans construct social space and the importance of this knowledge for social interaction (Hayduk, 1983; Argyle, 2013), little is known on how children represent social space and whether there are developmental differences between childhood and adulthood. The current study was designed to shed light on this question.

Young children do regulate their own personal space from early on. One prominent example is the strange situation test that is widely applied in research on attachment. In this test, infants’ active approach behavior to their caregiver is one central aspect of a secure attachment, whereas their avoidance of contact points to insecure attachment (Ainsworth et al., 1978). Interestingly, also in adults individual differences in the selection of interpersonal distance are related to their attachment security (Kaitz et al., 2004). Beyond the relevance of interpersonal space in attachment theory, further research has explored general trends in proxemic behavior in social interactions. In a seminal study, Aiello and Aiello (1974) paired 6- to 16-year-old participants with same-sexed peers and observed their proxemic behavior. The authors reported that children used more space the older they were. Likewise, Wesley and McMurphy (1982) observed 6-month-old to 5-year-old children’s positioning during free play interactions. They reported that children increased their distance from adult caretakers with age, while they decreased their distance to playmates. For school aged children, studies have reported an increase in distance to close friends (Burgess, 1981), although one study found this pattern only in groups of white children or mixed-race groups, but not in groups of black children (Willis et al., 1979; but see also Jones and Aiello, 1973). Assuming that younger children are more dependent on other persons than older children, the developmental findings might relate well to the adult literature. Here, it has been reported that priming of an independent self as well as greater independent self-construal leads to greater interpersonal distance (Holland et al., 2004). Interestingly, a recent study suggests that 4- to 5-year-old children’s proxemic behavior is affected by their need to belong. If children were primed with ostracism cues, they were shown to sit closer to a stranger (Marinovic et al., 2017). Overall, these studies demonstrate the regulation of interpersonal space in young children. In addition, they suggest an age-related trend for increasing interpersonal distance in the course of development.

A different question concerns how people in general and children in particular represent and reason about interpersonal space. That is, the mere fact of showing interpersonal space preferences does not clarify whether people in fact have a cognitive representation of interpersonal space or mainly display (potentially preconscious) behavioral preferences. Representing and reasoning about interpersonal space relates to an understanding and prediction of others’ behavior and could support fluent social interactions (e.g., Hayduk, 1983; Argyle, 2013) as well as efficient social cooperation (Sebanz et al., 2006; Vesper and Sebanz, 2016). It may help people to literally navigate the social world (cf. Banaji and Gelman, 2013).

Indeed, in the preschool years, children develop remarkable knowledge about the social world that helps them to reason about and predict others’ behavior. It has been shown that preschool children predict others’ actions based on their individual beliefs (Wimmer and Perner, 1983), past performances (Boseovski and Lee, 2006), and social rules (Bernard et al., 2016). In addition, with respect to physical space, preschool children predict an agent to take a shorter path to reach a goal, indicating a consideration of distances (Schuwerk and Paulus, 2016). From a theoretical point of view, these developmental achievements could be explained by the emergence and growth of representational abilities that allow preschool children to represent and reason about relations (Perner, 1991; Barresi and Moore, 1996). These emergent representational abilities should also allow preschoolers to represent and reason about social space.

Past research has shown that children do show some understanding of social space. Meisels and Guardo (1969) presented children from grades 3 to 10 with a booklet-based test in which they were presented with depictions of different persons, inter alia a friend and a stranger. They were asked to position a figure representing themselves on the sheet. Most relevant for the current study, the authors reported that younger children used greater spatial distances. It should be noted that this result stands in contrast to the reversed developmental trend (reported above) according to which children used more space the older they are (Aiello and Aiello, 1974; Wesley and McMurphy, 1982). More recently, Song et al. (2015) showed that, after being presented with videos depicting ostracism, 4- and 5-year-old children were more likely to draw pictures of themselves and their friends standing closer together. Given that these tasks were administered in a representational format, it indicates some understanding of interpersonal space. Yet, given that children depicted their own current situation, it remains open to which extent they possess a more generic understanding of interpersonal space. A more decisive line comes from research on the development of reasoning about friends. Preschool children have been shown to understand that close distances signify a friendly relationship by the preschool years (Post and Hetherington, 1974; Melson, 1976). Taken together, current and past research suggests that children do represent and reason about social space.

However, the findings do not paint a clear picture. For one, studies on age-related changes yield inconsistent findings. Whereas one study reported an increase in social distance across middle childhood (Burgess, 1981), did another study on children’s representation of social space revealed a reversed developmental pattern (Meisels and Guardo, 1969). Second, some studies using a representational task (e.g., puppets) included the child herself in the task (e.g., being represented by another puppet; Meisels and Guardo, 1969; being represented in a drawing; Song et al., 2015). Here, it remains unclear to which extent children’s responses rather demonstrates their personal experiences or wishes, or really demonstrate an agent-neutral understanding of social space. Thus, it remains controversial how children reason about social space and whether there are developmental changes.

The current study was designed to contribute to a clarification of these issues and to explore how children represent social space. More concretely, it examined the expectations children and adults have on how others occupy interpersonal space by employing a novel third-party paradigm. We decided to rely on an operationalization in which social space was assessed by means of distances in a sitting context. We thought that such a context might be familiar even to young children. Furthermore, we reasoned that the placement of distinct chairs would help children in selecting distances as it provides children with a clear anchor. Finally, sitting contexts have been successfully employed in recent studies on children’s (Marinovic et al., 2017) and adults’ own behavior (e.g., Camperio and Malaman, 2002; Novelli et al., 2010). The paradigm depicted an area in which four chairs (Experiment 1) or six chairs (Experiment 2) were placed in a rectangular array. One of the seats was already taken by an agent, when the protagonist entered the scene. Participants were asked to predict which of the remaining chairs the protagonist will select. Thus, we were able to examine whether participants expected the protagonist to choose a seat that was close to the stranger or a more distant seat. Given that our participants were not themselves involved in the paradigm (that is, were not represented in the task), but rather presented with figures depicting strangers, this paradigm helped us at investigating participants’ agent-neutral understanding of social space.

We selected preschool children as our youngest age group as preschool children have been suggested to possess the representational abilities that allow them to represent and reason about relations (Perner, 1991; Barresi and Moore, 1996) and as previous research has indeed provided first evidence that preschoolers represent social space (e.g., Meisels and Guardo, 1969). In addition, we selected a sample of adult participants to assess potential age-related differences. Current theories on the basis of social cognition indicated a strong link between children’s own action tendencies and their understanding of others (e.g., Goldman, 2006). Given the findings that children use more space the older they are (Aiello and Aiello, 1974; Wesley and McMurphy, 1982), we hypothesized that preschool children rather expect two persons to be closer to each other, whereas adults rather expect two persons to keep a greater interpersonal distance.

## Experiment 1

### Methods

#### Participants

The final sample consisted of 49 kindergarten children (mean age: 62.1 months; age range: 42.2–83.3 months; 23 male) and 20 adult participants (mean age: 23.7 years; age range: 18.2–29.1 years; 10 male). Three additional children were tested, but had to be excluded due to not finishing the experiment (*n* = 2) or failing a control question (*n* = 1). Child participants attended day care centers in a German city. Children were native German speakers from heterogenous socioeconomic backgrounds. Adult participants were recruited from a local student population and by means of worth. The study followed the ethical principles outlined by the Helsinki’s, 1964, declaration, but was not individually reviewed by an ethics committee given that formal ethical approval was not required at LMU Munich or by national laws at the time the research was conducted. Parents of participating children gave written and informed consent.

#### Procedure and Materials

**FIGURE 1:**
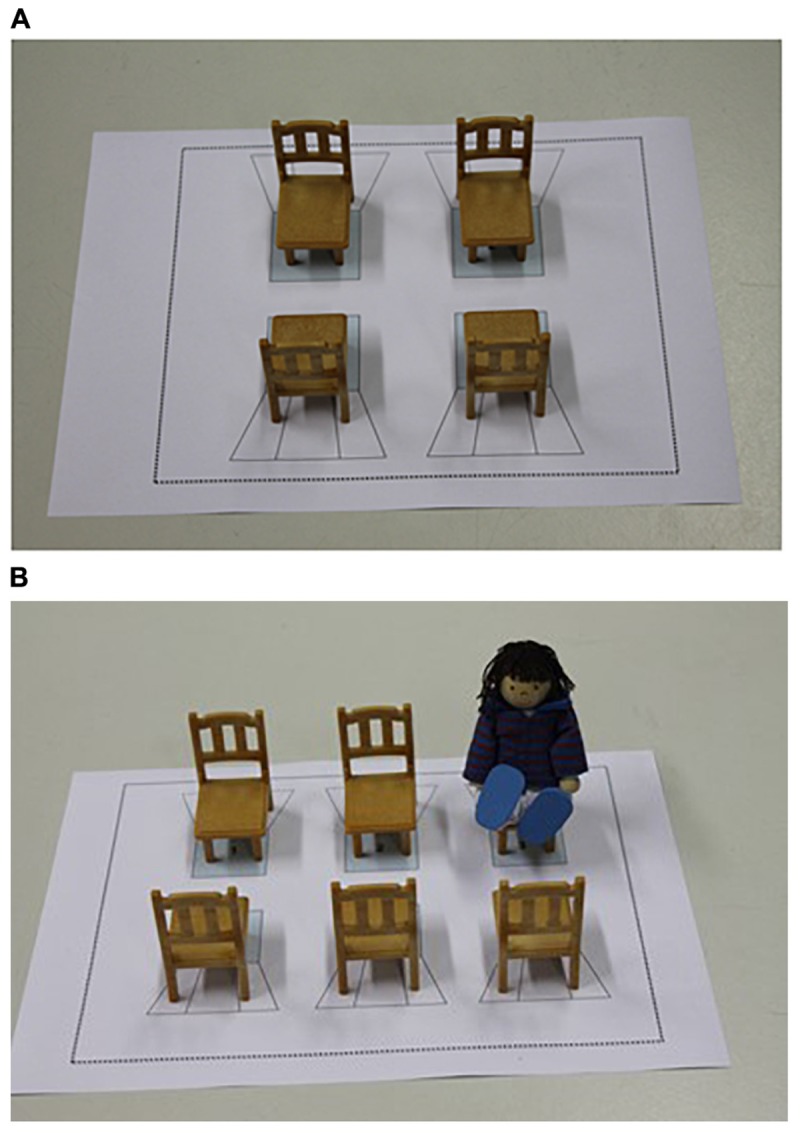
The figures display the experimental setups used in Experiment 1 **(A)** and Experiment 2 **(B)**.

Materials of the task consisted of five male and five female puppets (circa 10 × 6 cm). A piece of cardboard (DIN A4 sized) and four toy chairs (circa 6 × 3.5 cm) created from cardboard served as a train cabin. The chairs were positioned in a rectangular assembly (circa 5 × 6 cm) on the cardboard (see Figure 1A). A camera was used to record the test sessions.

Participants were tested individually in a quiet room. Experimental sessions were videotaped and scored online by the experimenter. Participants were first familiarized with the protagonist puppet (who was randomly selected from the five puppets that matched the child’s gender). The protagonist was introduced by name (e.g., Lea). Subsequently, the experimenter introduced the train cabin. To this end, the experimenter connected the area with the child’s personal experiences. For example, she pointed out that when one travels by train or tram one can sit down in a cabin with several seats, and that the current area (depicted by the cardboard and the chairs) represents such a cabin in which the seats allow people to sit down. One child was not able to follow the instructions and was therefore excluded.

Next, we administered a familiarization trial in which children were familiarized with the procedure of seating the protagonist on one of the chairs. In particular, children were told that the protagonist wants to travel by train and take a seat. Participants were asked to guide the protagonist to one of the seats. All children were able to do so. Thereafter, the protagonist was removed.

The test phase consisted of eight trials. All test trials followed the same structure. The experimenter told the child that another person wants to take the train, enters the compartment, and takes a seat. No clue to the age of the person was given. While explaining, the experimenter placed one of the other puppets at one of the chairs. Subsequently, the experimenter continued: “Lea (the protagonist) wants to take the tram and take a seat. She realizes that there is already another person sitting on one of the chairs. Now she wants to take a seat as well. What do you think: On what chair is Lea going to take place?” The experimenter handed over the protagonist puppet to the participant so that s/he could place the protagonist on a seat. We balanced on which of the four chairs the other person was seated between trials and the order of trials between participants.

#### Coding and Data Analysis

We analyzed as dependent variable at which of the three remaining seats participants placed the protagonist. We analyzed the data by a 2 (age groups: children, adults) × 3 (chair positions: next, opposite, diagonal) mixed-model analysis of variance (ANOVA). A preliminary analysis including the factor gender revealed no significant effect of gender so that we dropped this factor from further analyses. In addition, correlational analyses with age were conducted for the preschool sample to assess potential age-related changes.

### Results

**FIGURE 2:**
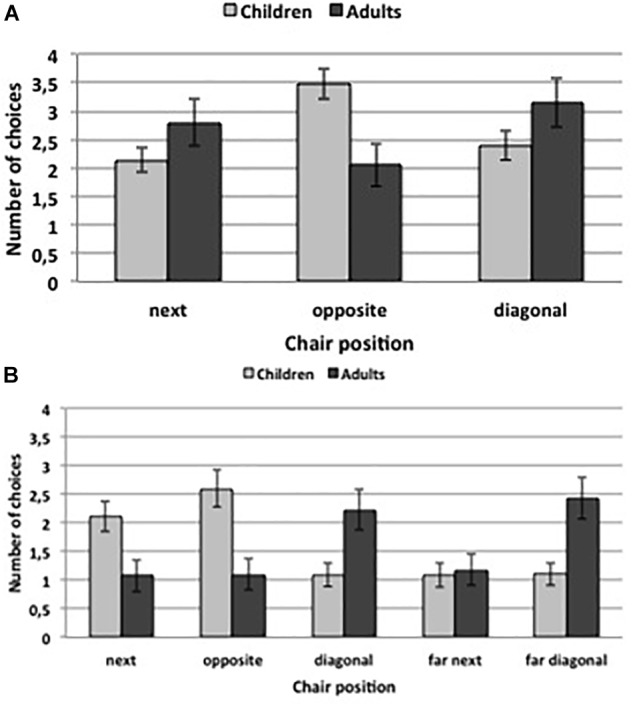
Average number of trials in which participants choose the respective chair position. **(A)** shows the results of Experiment 1 and **(B)** the results of Experiment 2. Error bars indicate standard errors of the means.

Figure 2A shows the mean number of trials in which participants chooses the different seats (see also Table 1A). Although data were non-normally distributed analysis of variance procedures are known to be robust to violations of normality (e.g., Schmider et al., 2010); therefore, analyses were conducted as planned. The ANOVA revealed a significant interaction effect of age group and chair position, *F*(2, 134) = 4.81, *p* = 0.014, η_p_^2^ = 0.07 (all other *p*s > 0.69). To follow-up on the interaction effect, planned paired samples *t*-tests were conducted for each age group. For preschool children, the tests revealed that they chose more often the opposite position than the next position, *t*(48) = 3.31, *p* = 0.002, as well as more often the opposite than the diagonal position, *t*(48) = 2.37, *p* = 0.022. There was no difference between next and diagonal position, *t*(48) = 0.63, *p* = 0.530. There was no significant effect for adult participants (all *p*s > 0.13).

**Table 1:** Means and standard errors (presented in brackets) of the dependent variables in Experiment 1 **(A)** and Experiment 2 **(B)**.

	Next	Opposite	Diagonal		
**(A) Experiment 1**					
Children	2.14 (0.21)	3.47 (0.26)	2.39 (0.25)		
Adults	2.80 (0.41)	2.05 (0.37)	3.15 (0.44)		

	**Next**	**Opposite**	**Diagonal**	**Far next**	**Far diagonal**

**(B) Experiment 2**					
Children	2.11 (0.26)	2.60 (0.32)	1.09 (0.20)	1.09 (0.21)	1.11 (0.20)
Adults	1.08 (0.28)	1.10 (0.27)	2.23 (0.36)	1.18 (0.27)	2.43 (0.36)

In addition, independent samples *t*-tests revealed that children were more likely to choose the opposite chair than adults, *t*(67) = 3.04, *p* = 0.003. There was no significant effect on the adjacent position or diagonal position, *t*(67) = 1.54, *p* = 0.128, and *t*(67) = 1.59, *p* = 0.116, respectively.

We also analyzed whether position selection was related to child age. To this end, we calculated correlational analyses between the number of each of the chair position choices and child age. There was no correlation with age (all *r*s < 0.15, all *p*s > 0.30).

### Discussion

Experiment 1 aimed at examining preschool children’s and adults’ prediction about others’ interpersonal distance preferences. It revealed two important findings. First, preschool children’s predictions were not at random. Rather, they showed a systematic prediction pattern. Preschool children expected the protagonist to choose a chair that was close (here: opposite) to the other person. This is partly in line with our hypothesis as children chose this seat more often than adults did. Yet, given that there was no such preference for the chair next to the protagonist, the results were not fully in line with our hypothesis. Second, there was an age-related difference as adults were less likely to choose the opposite chair compared to children. However, in contrast to our predictions there was no systematic preference for one chair in the adult sample.

One possibility is that the circumscribed sitting area with only four seats might not allow for the detection of greater interpersonal distance preferences in adults given that all chairs were quite close to each other. A set-up in which some chairs are more distant from each other might thus be more suitable. Thus, before drawing theoretical conclusions, we wanted to explore this possibility in another experiment. Running a second experiment would furthermore also allow us to examine whether we could replicate the findings from Experiment 1.

To this end, we designed Experiment 2. This experiment closely followed Experiment 1 with the crucial difference that we employed six chairs rather than four chairs. Three chairs were positioned in a row and the two rows were facing each other. As a consequence, there was a greater distance between the chairs that were placed at the corners of each row. We hypothesized that preschool children would expect the protagonist to choose a seat close to the stranger, most likely the opposite one, whereas adults would expect the protagonist to choose a distant seat to the stranger, most likely the far diagonal one.

## Experiment 2

### Methods

#### Participants

The final sample consisted of 35 kindergarten children (mean age: 57.2 months; age range: 42.2–73.2 months; 16 male) and 40 adult participants (mean age: 26.1 years; age range: 19.2–54.0 years; 17 male) who did not take part in Experiment 1. Child and adult participants came from the same population as in Experiment 1. As in Experiment 1, the study followed the ethical principles outlined by the Helsinki’s, 1964, declaration, but was not individually reviewed by an ethics committee given that formal ethical approval was not required at LMU Munich or by national laws at the time the research was conducted. Parents of participating children gave written and informed consent.

#### Procedure and Materials

Materials and procedure of Experiment 2 followed Experiment 1 with the difference that the cabin area on the cardboard consisted of six chairs. There were two rows with three chairs each facing each other (see Figure 1B). The adjacent and opposite chairs were placed in the same distance from each other (ca. 4.5 cm). The waiting puppets were placed in one of the four chairs at the corner positions so that children could lead the protagonist puppet to one of the following five free chairs: adjacent, opposite, diagonal, far adjacent, far diagonal. Eight test trials were administered. We balanced between trials at which of the four seats the other person was seated and between participants the order of trials.

#### Coding and Data Analysis

We analyzed at which of the five remaining seats participants placed the protagonist. We analyzed the data by a 2 (age groups) × 5 (chair positions) mixed-model ANOVA. A preliminary analysis including the factor gender revealed no significant effect of gender so that we dropped this factor from further analyses. In addition, correlational analyses with age were conducted for the preschool sample to assess potential age-related changes.

### Results

Figure 2B shows the mean number of trials in which participants chooses the different positions (see also Table 1B). Even though data were non-normally distributed analysis of variance procedures are known to be robust to violations of normality (e.g., Schmider et al., 2010), therefore analyses were conducted as planned. The ANOVA revealed a significant interaction effect of age group and chair position, *F*(4,292) = 7.86, *p* < 0.001, η_p_^2^ = 0.10 (all other *p*s > 0.18). To follow-up on the interaction effect, paired samples *t*-tests were conducted for each age group (see Table 2). Overall, the analyses showed that children were more likely to choose the next and the opposite chair, whereas adults were more likely to choose the diagonal and the far diagonal than the others.

**Table 2:** Paired samples *t*-tests following up on the significant interaction of age group and chair position in Experiment 2.

	Children		Adults	
	*t*	*d.f.*	*t*	*d.f.*
Next – opposite	-1.10	34	-0.06	39
Next – diagonal	2.80^∗∗^	34	-2.69^∗^	39
Next – far diagnonal	2.74^∗^	34	-2.40^∗^	39
Next – far next	2.64^∗^	34	-0.23	39
Opposite – diagonal	3.40^∗∗^	34	-2.14^∗^	39
Opposite – far diagonal	3.37^∗∗^	34	-2.62^∗^	39
Opposite – far next	3.40^∗∗^	34	-0.20	39
Diagonal – far diagonal	-0.10	34	-0.34	39
Diagonal – far next	0.00	34	1.91^∗^	39
Far diagonal – far next	0.10	34	2.66^∗^	39

^∗^*p < 0.05, ^∗∗^*p* < 0.01. See Figure 2B for means*.

Independent samples *t*-tests revealed that children were more likely than adults to choose the next chair, *t*(73) = 2.72, *p* = 0.008, and the opposite chair, *t*(73) = 3.64, *p* = 0.001, whereas adults were more likely to choose the diagonal chair, *t*(73) = 2.68, *p* = 0.009, or the far diagonal chair, *t*(73) = 3.02, *p* = 0.004. There was no difference with respect to the far next chair, *t*(73) = 0.25, *p* = 0.800.

We also analyzed whether position selection was related to child age. To this end, we calculated correlational analyses between the number of each of the chair position choices and child age. There was no correlation with age (all *r*s < 0.19, all *p*s > 0.27).

### Discussion

The results of Experiment 2 demonstrated that children were more likely to place the protagonist at one of the chairs close to the stranger, whereas adult predicted that the protagonist would choose a seat in a more distant position, most notably in a diagonal or far diagonal position. This pattern is in line with our hypothesis of an age-related increase in children’s reasoning about interpersonal space. The results will be discussed in the following section.

## General Discussion

The current study aimed at examining preschool children’s and adults’ reasoning about interpersonal space, and at investigating potential age-related differences. Two experiments provided evidence that preschoolers rather expected two strangers to take positions that are closer to each other, while adults rather expected them to stay in greater distance. This study confirms that by the preschool years, children have systematic predictions about interpersonal space. Moreover, it suggests developmental changes in people’s representation of social space with adults assuming people to keep greater interpersonal space than preschool children.

Notably, our findings on age-related differences in people’s representation of interpersonal space relate to work about humans’ own proxemics in social interactions. Here, it has been reported that with increasing age people need more personal space and keep more distance from strangers (e.g., Burgess, 1981). This relates well to a study on personal space in adult urban passenger train commuters. It was found that people experienced aversive reactions when they had to sit close to others (Evans and Wener, 2007). Given the parallels in the developmental trend, these result patterns suggest that participants’ predictions of others’ behavior may be based on their own action tendencies. That is, our findings are in line with theoretical proposals that people use their own action tendencies and experiences to predict others’ behavior (e.g., Goldman, 2006) and extend this research program to the area of people’s reasoning about interpersonal space.

We offer two (not mutually exclusive) interpretations for the difference between children and adults. First, young children are on average more vulnerable than adults and depend to a greater extent on others. One could thus argue that they are more used to stay in close contact to others or seek contact to others instead of staying alone. Second, children might find more ease in approaching others and therefore expect similar behavior from others, whereas adults have acquired rules of how to behave toward strangers, most notably not to invade others’ personal space and to keep appropriate distances (Hayduk, 1983; Argyle, 2013). Conversely, it is reasonable to assume that they also expect others to follow these rules. Indeed, empirical research demonstrated that with increasing age, children are more insistently instructed to keep appropriate distances (Fry and Willis, 1971). The combination of both factors could explain the difference in children’s and adults’ reasoning about interpersonal space as observed in the current study.

It should be noted that our results were not in line with findings by Meisels and Guardo (1969) who reported that children used less space the older they were. The authors used a semi-projective task in which children were presented with drawings of persons on paper sheets. The drawings were said to represent different persons, inter alia the participants’ best friend or a stranger. One cutout figure represented the participant herself. Participants were instructed to place the cutout figure on the paper sheets so that the distance between the figure and each drawing could be assessed. One difference between this task and the current one is that Meisels and Guardo (1969) used a semi-projective measure in which participants represented themselves by means of a cutout figure. Yet, projective measures might be problematic as it is not clear what exactly is assessed in these tasks. Notably, Love and Aiello (1980) compared adult participants’ actual proxemic behavior in a real interaction with their responses in three projective tasks. Neither of the three tasks was related to participants’ proxemics in the real-life task, questioning thus the validity of projective measures for an assessment of personal distance. It is thus possible that the findings of Meisels and Guardo (1969) do neither represent participants’ own interaction distance preference (cf. Aiello and Aiello, 1974; Wesley and McMurphy, 1982) nor their reasoning about third parties (as in our task), but rather different processes (e.g., how they would prefer to interact with others). Regardless of the explanation for this divergent finding, our study contributed to a clarification of the question how people represent social space and how it changes with age. The current study may thus lay the foundation for further work using social space as a measure to investigate early socio-emotional and social-cognitive development (e.g., Song et al., 2015; Marinovic et al., 2017) as it reveals developmental changes in humans’ conception of social space.

The current study also has limitations and leaves some open questions for further research. First, following previous research on the development of social space (e.g., Marinovic et al., 2017) and representational abilities (e.g., Perner, 1991), the current study focused on the preschool period to assess developmental differences. Given that no relationship between age and expectations about interpersonal space was found within the preschool sample, it would be particularly interesting to assess elementary school children’s and adolescents’ expectations about social space. Second, it would be highly interesting to investigate the factors that impact children’s predictions of others’ interpersonal space further. For example, a recent study by Marinovic et al. (2017) demonstrated that children are more likely to approach a stranger after being confronted with ostracism cues. It would be interesting to see whether and from which age children also expect someone else to seek more contact after being exposed to ostracism. Such knowledge would allow children to adequately react to others’ needs and show efficient other-oriented comforting behaviors. Third, the current study operationalized interpersonal space in a sitting context. It would be interesting to explore whether the same pattern of results would be obtained when relying on other operationalizations. We have to leave it to future research to address this question. Fourth, since we presented participants with several trials, we decided to not include follow-up questions and probe their reasoning behind their choices. This decision was based on findings that young children tend to interpret repeated “why”-questions as a sign that they did something wrong and should change their behavior (cf. Siegal, 2005). It would be interesting to explore children’s explicit justifications in a future study. Finally, there is plenty of evidence that proxemics strongly differ between cultures (Hall, 1966; Watson, 1970). The current study was conducted in a Western culture in which people usually keep greater interpersonal distance. Yet, this culture is not prototypical (Henrich et al., 2010). People in more collectivist and non-Western cultures have been shown to prefer closer interpersonal space (Hall, 1966). It would be interesting to explore whether there are similar cross-cultural differences in people’s reasoning about social space and to which extent people are aware of cross-cultural differences in others’ interpersonal space preference. The acquisition of such knowledge could be an important aspect of intercultural competencies. We have to leave it to future research to address these questions and to shed further light on how people represent interpersonal space.

## Data Availability Statement

The data used in this study is available in Supplementary Datasheet 1.

## Author Contributions

The author confirms being the sole contributor of this work and has approved it for publication.

## Conflict of Interest Statement

The author declares that the research was conducted in the absence of any commercial or financial relationships that could be construed as a potential conflict of interest.
